# Development assistance, donor–recipient dynamic, and domestic policy: a case study of two health interventions supported by World Bank–UK and Global Fund in China

**DOI:** 10.1186/s41256-024-00344-3

**Published:** 2024-02-04

**Authors:** Aidan Huang, Yingxi Zhao, Chunkai Cao, Mohan Lyu, Kun Tang

**Affiliations:** 1 Vanke School of Public Health, Tsinghua University, No. 30 Shuangqing Road, Beijing, 100084 China; 2 Institute for International and Area Studies, Tsinghua University, Beijing, China; 3 Department of Medicine, University of Oxford, Nuffield Oxford, UK; 4 Duke Global Health Institute, Duke University, Durham, NC USA

**Keywords:** Development assistance for health, Sustainability, Transition, Donor–recipient dynamic, Health policy, China, World Bank, DFID, Global Fund to Fight AIDS, Tuberculosis and Malaria

## Abstract

**Background:**

This study views sustainability after the exit of development assistance for health (DAH) as a shared responsibility between donors and recipients and sees transitioning DAH-supported interventions into domestic health policy as a pathway to this sustainability. It aims to uncover and understand the reemergent aspects of the donor–recipient dynamic in DAH and how they contribute to formulating domestic health policy and post-DAH sustainability.

**Methods:**

We conducted a case study on two DAH-supported interventions: medical financial assistance in the Basic Health Services Project supported by the World Bank and UK (1998–2007) and civil society engagement in the HIV/AIDS Rolling Continuation Channel supported by the Global Fund (2010–2013) in China. From December 2021 to December 2022, we analyzed 129 documents and interviewed 46 key informants. Our data collection and coding were guided by a conceptual framework based on Walt and Gilson’s health policy analysis model and the World Health Organization’s health system building blocks. We used process tracing for analysis.

**Results:**

According to the collected data, our case study identified three reemergent, interrelated aspects of donor–recipient dynamics: different preferences and compromise, partnership dialogues, and responsiveness to the changing context. In the case of medical financial assistance, the dynamic was characterized by long-term commitment to addressing local needs, on-site mutual learning and understanding, and local expertise cultivation and knowledge generation, enabling proactive responses to the changing context. In contrast, the dynamic in the case of HIV/AIDS civil society engagement marginalized genuine civil society engagement, lacked sufficient dialogue, and exhibited a passive response to the context. These differences led to varying outcomes in transnational policy diffusion and sustainability of DAH-supported interventions between the cases.

**Conclusions:**

Given the similarities in potential alternative factors observed in the two cases, we emphasize the significance of the donor–recipient dynamic in transnational policy diffusion through DAH. The study implies that achieving post-DAH sustainability requires a balance between donor priorities and recipient ownership to address local needs, partnership dialogues for mutual understanding and learning, and collaborative international–domestic expert partnerships to identify and respond to contextual enablers and barriers.

**Supplementary Information:**

The online version contains supplementary material available at 10.1186/s41256-024-00344-3.

## Background

Donors in development assistance for health (DAH) play a significant role in shaping health policies in many low- and middle-income countries [1–9]. They exert their influence through financial resources, knowledge transfer, technical expertise, inter-sectoral leverage, and indirect financial and political incentives [5, 6, 10, 11]. Factors such as colonial legacies [5, 12–15], donors’ concerns over domestic bureaucratic capacity, and domestic elites’ vested interests gained from the past DAH could contribute to the weak bargaining power of domestic policy actors [13, 15–19]. Donors’ influence, therefore, could align domestic actors and institutions with donor-dominated systems [20–23], leading to distortions in healthcare systems and exacerbating health inequalities [20, 24–28].

However, the recipient’s agency plays a role. Whether aid-dependent or not, domestic actors can portray DAH as instrumental in validating and legitimizing local policy agendas [29–33]. They strategically engage with donors [22, 28, 34] and act as proactive gatekeepers, brokers, translators, and innovators [12, 22], demonstrating variable levels of ownership in leveraging access to DAH resources [34–36]. Specifically, they emphasize contextual differences and selectively frame or reformulate externally transplanted policies [7, 28, 37–40]. Consequently, the local adaptation of foreign ideas, involving continuous socio-political interactions among domestic and external actors, is widely recognized as key for transnational policy diffusion [22, 40–44].

Therefore, the relationship between donors and recipients is not unidirectional [41, 42, 45]. It involves mutual interaction, negotiation, and compromise [9, 18, 20, 34, 42]. For example, donors can socialize and empower domestic brokers in response to the recipient’s agency, forming alliances for policy change [31, 33, 35]. However, while frameworks [12, 21] and typologies [14] exist for analyzing donors’ influence and recipients’ agency in domestic health policy formulation, few studies have unpacked the complexity of donor–recipient dynamic in DAH and the dynamic’s influence on domestic health policy formulation.

This study aims to uncover the reemergent aspects of this dynamic and understand how they interact and construct causal mechanisms, linking this dynamic with domestic health policy formulation. It claims that maintaining or scaling up DAH-supported interventions does not guarantee sustainability, especially when they undermine local capacity or distort priorities in addressing health inequities [28, 46, 47]. Instead, post-DAH sustainability should be a shared responsibility between donors and recipients for improving health outcomes and reducing health inequities [48–50]. Transitioning context-based DAH-supported interventions into domestic health policy, as one form of transnational policy diffusion, could thus be a pathway to this sustainability. The study does not assume that DAH is apolitical [51]. Rather, it suggests that with more resources and a broader scientific network, DAH donors can effectively contribute to domestic policy formulation and post-DAH sustainability while achieving strategic interests by continuously interacting with recipients [52, 53]. In other words, the donor–recipient dynamic can reach a point where DAH maximizes sustainable health outcomes.

We conducted a case study in China to explore this dynamic, focusing on two interventions initially supported by two DAH projects (Table 1). Neither of the two interventions had been part of national policies before the implementation of these selected projects. One is medical financial assistance (MFA) in the Basic Health Services Project (BHSP) supported by the World Bank (hereinafter referred to as ‘the Bank’) and UK DFID (now the Foreign, Commonwealth and Development Office), from 1998 to 2007. Another is civil society engagement in the HIV/AIDS Rolling Continuation Channel (RCC) supported by the Global Fund to Fight AIDS, Tuberculosis and Malaria (hereinafter referred to as ‘the Fund’), from 2010 to 2013. The two cases share similarities in funding levels and geographical coverage, with the involved donors being the largest in supporting MFA and HIV/AIDS civil society engagement in the country, respectively. However, they have shown varying impacts on domestic health policy formulation and post-DAH sustainability.

**Table 1:** Selected cases

Case	Basic information	Contribution to domestic health policies
Medical financial assistance in the Basic Health Services Project supported by the World Bank, DFID, and other donors (1998–2007)	This project focused on 97 impoverished rural counties in ten underdeveloped provinces in China, targeting around 43 million people. DFID supported the BHSP through a Health 8 Support Project with a grant equivalent to 16% of total project costs. The Ministry of Health was in charge of implementing the project. One of the project’s components is the medical financial assistance scheme. This scheme partially reimbursed providers for healthcare services and inpatient care within participating townships. Specifically, it covered the poorest 5% of households in 71 counties and the poorest 20% of households in additional 26 counties	The medical financial assistance scheme implemented by this project was the first large-scale experiment in China aimed at establishing a health safety net for impoverished populations. It has proved to be one of the significant drivers in shaping the initial policy formulation on national rural medical assistance around 2003 and subsequently contributed to the refinement of this policy during the national healthcare reform in 2009. The national medical assistance program has now been integrated into the social assistance scheme implemented by the Ministry of Civil Affairs. Institutionalized throughout the country, it achieved broader and more effective coverage than the project
Civil society engagement in the HIV/AIDS Rolling Continuation Channel supported by the Global Fund to Fight AIDS, Tuberculosis and Malaria (2010–2013)	The Global Fund has been the foremost and enduring donor for HIV/AIDS and played a pivotal role in fostering the growth of HIV/AIDS civil society organizations in China since 2003. The Rolling Continuation Channel was the last Global Fund grant that consolidated previous rounds, integrating international and domestic resources to ensure program sustainability. The project, spanning 31 provinces in China, employed a multisectoral governance mechanism and promoted institutional capacity building for HIV/AIDS civil societies. Chinese Center for Disease Control and Prevention was the Principal Recipient of the project	The project provided a model of civil society engagement and HIV/AIDS program management for China’s HIV/AIDS response, leaving significant legacies for the post-transition programs, China AIDS Fund for Non-Governmental Organizations and subnational government contracting. However, it failed to fully strengthen HIV/AIDS civil society organizations in China for their sustainable post-transition operations amid government dominance. While post-transition management has been stricter and improved, they primarily focus on target-oriented testing and treatments. Survived civil society organizations have largely become “passive helpers” constrained by government requests

Source: [54–57]

## Methods

### Study design

The research question of this study is how the donor–recipient dynamic in DAH influences the formulation of domestic health policy associated with DAH-supported interventions. We employed the case study approach to thoroughly examine complex systems [58], specifically comparing BHSP’s MFA and HIV/AIDS RCC’s civil society engagement in China. Our data collection took place from December 2021 to December 2022, during which we extensively investigated 129 published literature articles, policy papers, and project documents and conducted in-depth, semi-structured interviews with 46 key informants. To guide data collection and coding, we utilized a conceptual framework (hereinafter referred to as ‘policy triangle framework’) rooted in Walt and Gilson’s health policy analysis model [59] and the World Health Organization’s health system building blocks [60]. Finally, we adopted the method of process tracing [61] in within-case analysis and conducted cross-case analysis.

### Conceptual framework

**Fig. 1:**
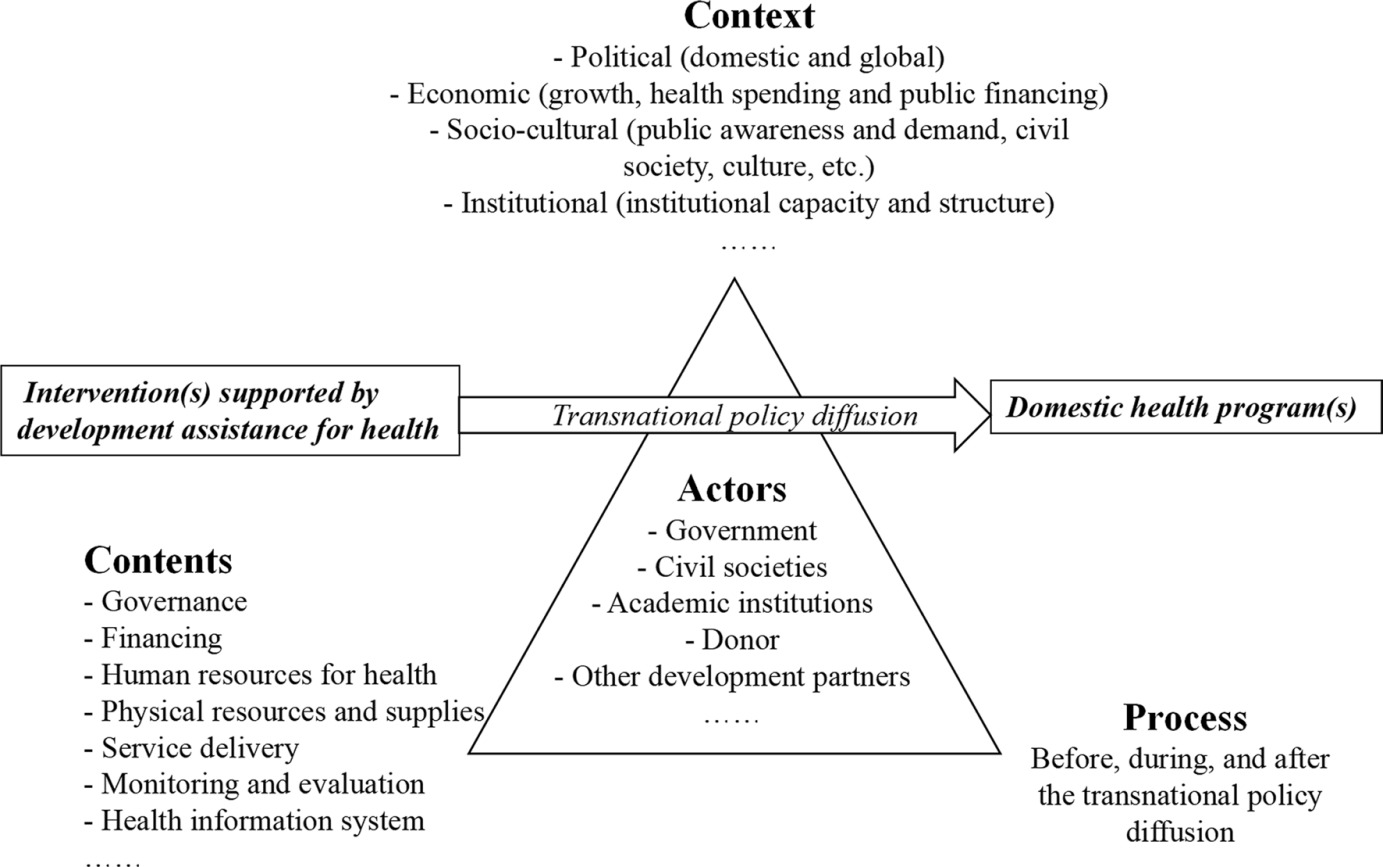
Conceptual framework

The conceptual framework of this study is adapted from the policy triangle framework of a cross-country research project supported by the Alliance for Health Policy and Systems Research, World Health Organization, the Department of Health Systems Governance and Financing, World Health Organization, and UHC 2030, called “sustaining effective coverage in the context of transition from external assistance—lessons from countries” [62]. Based on China’s situation, the adapted policy triangle framework in this study (Fig. 1) aims to examine how DAH-supported interventions transition into domestic health programs within the local health system. This framework aligns with Walt and Gilson's health policy analysis model [59], which consists of four key aspects: context, content, actor, and process. The World Health Organization’s health system building blocks form the basis of ‘content’ [60], as these blocks are critical to analyze health system interventions by function.

Specifically, our framework explores the interplay among the following elements: context (political, economic, institutional, and socio-cultural factors), content (governance, financing, service delivery, etc.), actors (government, donors, civil societies, etc.), and processes occurring before, during, and after the transnational policy diffusion. Within this framework, we aimed to identify both enablers and barriers to transnational policy diffusion through DAH, drawing insights from the aspects of ‘context’, ‘content’, and ‘actors’. The ‘process’ element further elucidates how these enablers and barriers manifest and exert their influence.

To address our research question, we narrowed our focus within the policy triangle framework to the ‘actors’ aspect, specifically donors and recipients, along with their interactions. Instead of strictly separating the four aspects of the framework during data analysis, we prioritized examining the donors, recipients, and their interactions within the ‘process’ of transnational policy diffusion. This process operates through the ‘content’ of DAH-supported interventions within the specific ‘context’ of each case. Here, ‘donor’ refers to external actors who transfer resources for DAH, including the funders, managing agencies, and external experts involved in the disbursement and utilization of the transferred resources. This definition distinguishes ‘donor’ from ‘development partner’, which includes actors providing technical support without being commissioned for DAH. The term ‘recipient’ encompasses domestic counterparts receiving and utilizing transferred resources for DAH, extending beyond the government to include technical agencies, civil societies, beneficiary populations, and other relevant parties. It is important to note that this study recognizes diverse approaches and behaviors of actors within the donor and the recipient. However, as this study primarily investigates donor–recipient dynamic, it does not delve into the internal interactions of the donor or the recipient.

### Data collection

We conducted both primary and secondary data collection.

Secondary data were obtained through a scoping review. This review encompassed 129 diverse sources, including research articles, research reports, policy briefs, project documents and reports, and national policies or strategies relevant to the cases, covering the period from 1997 to 2022. The search strategy combined terms and phrases related to the World Bank, DFID, and the BHSP, as well as Global Fund and HIV/AIDS. We then conducted searches using various databases such as Embase, MEDLINE, and China National Knowledge Integrated Database. Additionally, we performed supplementary searches on platforms, including Google, Google Scholar, World Bank Open Knowledge Repository and eLibrary, the Overseas Development Institute, and the official websites of the World Bank, the UK government, the Chinese Center For Disease Control And Prevention (CDC), and the Global Fund. In addition to these initial sources, we employed a snowballing approach by reviewing reference lists of related literature and conducting online searches to identify additional studies or documents. Some studies or documents were obtained through personal correspondence. Through careful screening of titles, abstracts, and full texts, we selected 64 studies and documents for the BHSP case and 65 for the RCC case. The data were then sorted under the policy triangle framework, yielding preliminary review findings that informed our interviews. For a detailed account of the scoping review process and the major project and policy documents, please refer to Additional file 1. The scoping review report followed the Preferred Reporting Items for Systematic Reviews and Meta-analyses (PRISMA) methodology for scoping reviews [63].

To collect primary data, we conducted in-depth semi-structured interviews. Based on the scoping review, we employed a purposive sampling technique to select key informants from various perspectives, including donor agencies, government organizations, civil societies, and academic institutions. We sent interview invitations to these identified key informants and utilized a snowballing approach whereby existing informants were asked to suggest additional key informants who could provide valuable insights. Out of the 85 informants contacted, 46 participated in the interviews. Among these respondents, two were involved in both cases, 20 were associated with BHSP only, and 24 were associated with RCC only. Detailed profiles of each respondent can be found in Additional file 1, while a summary of the respondents’ profiles is provided in Table 2.

**Table 2:** A summary of the respondents’ profiles

Case project	Level	Number	Type of affiliation	Number	Engagement	Number
BHSP	International	5	Government	13	Project implementation only	8
	National	5	Donor or development partner	5	Project implementation and post-transition program implementation	14
	Subnational	12	Academic institution	2		
			Health center	2		
RCC	International	2	Government	3	Project implementation only	5
	National	12	Donor or development partner	2	Post-transition program implementation only	1
	Subnational	12	Academic institution	3	Project implementation and post-transition programs implementation	20
			Civil society organization	18		

In developing the interview guides, we initially structured the questions according to the policy triangle framework and then refined these questions based on the scoping review findings to better suit the specific contexts of the two cases. The prompts and questions were further modified iteratively based on insights gained from previous interviews and the background analysis of each informant. As a result, the interview guides differed between cases and individuals, allowing for open and in-depth discussions. The interviews had a duration ranging from 90 to 150 min. After each interview, we engaged in discussions to identify key findings and insights. Interviews were transcribed in the language they were conducted in, with four interviews in English and the rest in Chinese. To ensure accuracy, another team member validated the transcripts.

### Data analysis

Using MAXQDA 2022 and triangulating data from different sources, the data analysis process involved two stages using a framework approach [64, 65]. In the first stage, two coders conducted a pilot coding of ten interview transcripts to test major themes in the policy triangle framework and develop a coding scheme. The formal coding covered all data sources, inductively identifying recurrent themes and sub-themes and refining codes for each case. After this stage, we framed the research question of this study.

In the second stage, we employed process tracing to reorganize the codes and examine the evidence surrounding alternative explanations and policy outcomes. Process tracing is a qualitative research method for examining evidence surrounding alternative explanations and seeking to detect the mechanisms that link presumed causal factors with the policy outcomes of interest [42, 61, 66]. We first omitted codes identified as irrelevant to transnational policy diffusion (e.g., project procurement only for project implementation). Further, the analysis highlights the salience of ‘actors’ while maintaining the meaningful and interrelated aspects of ‘contents’, ‘context’, and ‘processes’ in the policy triangle framework. Cross-case analyses followed within-case analyses to identify similarities and differences across the cases, through which we induced three overarching themes of donor–recipient dynamics: different preferences and compromise, partnership dialogues, and responsiveness to the changing context.

### Ethical considerations

This study received ethical approval from the Institutional Review Board of Tsinghua University (Project No: 20210095). Before the interviews, all the respondents signed the written informed consent forms.

## Results

### Medical Financial Assistance supported by the World Bank and UK (1998–2007)

The context of sustaining MFA of the BHSP was challenging yet promising. According to respondents and project documents, while MFA was considered an embodiment of the Bank’s and DFID’s global pro-poor vision [67], it faced difficulties due to the marketization of the health sector since the late 1970s that dismantled the previous health safety nets in the planned economy era, according to respondents. However, the vague nature of China’s 1997 public health reform and the government’s general poverty reduction policies created an opportunity for implementing MFA [55, 67]. As China’s conservative domestic policy-making prioritized social stability and the avoidance of errors [67], the government saw external assistance as a chance to test, adapt, and refine new policies informed by experiences in other settings [56, 68], with MFA supported by the World Bank and the UK as part of this process.

#### Donors’ insistence on the need-based approach

The Bank and DFID emphasized their roles in the project design, insisting on the need-based approach embodied in the design of the project MFA. According to respondents, the Chinese government opposed using 25% of the Bank credits to finance the “intangible”, “uncertain” MFA (*R3-WB and R5-WB*), without prior policy experience [55, 69, 70]. Instead, they preferred to use these credits for what they perceived as “more sustainable” (*R3-WB*)—health facility construction, leading to a near breakdown in negotiations for project design. However, through informal discussions among key health economics experts from the Bank, Chinese experts, and managers, the parties reached a consensus on the importance of testing a government-endorsed medical assistance scheme. As a compromise, MFA was reduced to accounting for 5% of the overall amount and financed by Chinese counterpart funds [69].

“We argued a lot with the government during the project preparation because the government was unwilling to pay for this [medical assistance], taking into account the project’s sustainability when it was over. But we were insistent and firmly believed this was important for China. We then undertook extensive communication efforts to convey the necessity of this demand-side financial reform.” (R3-WB)

The donors’ insistence in the inclusion of MFA was regarded as “farsighted” [55] and realized through donor–recipient collaborative efforts. Supported by the government and local experts, the Bank and DFID conducted studies on the health sector’s needs and the complex context of the country, informing their project design and investment decisions [55, 70]. Domestic experts recalled that the Bank and DFID experts conducted multiple field visits in poverty-stricken areas and actively communicated with the local officials and experts to understand health needs and financial gaps [70]. In addition, having identified the bottlenecks in China’s rural health, the Bank had even tested the idea of MFA in a Bank’s previous maternal and child health project on a smaller scale in China, aiding in understanding MFA implementation [71].

Overall, the respondents described the donors as believing in long-term investment and viewing reforms as an evolutionary process, and this insistence took effect. Respondents noted that despite initial dissatisfaction with a report supported by the Bank criticizing the country’s health financing system around 1995, the government ultimately recognized the emerging health issues and the importance of reform, leading to a positive gesture—the 1997 public health system reform. This reform subsequently paved the way for implementing MFA as a policy pilot:

“In 1997, when the government decided to reform the country’s public health system, the BHSP seamlessly became a national policy pilot instead of swimming against the current, resulting from extensive sector studies carried out over a prolonged period. It is important to note that the Bank’s health projects were not initiated based on the governmen’'s request, but rather as a response to identified needs.” (R5-WB)

#### “Recipient in the driving seat”

“Recipient in the driving seat” (*R8/9-WB*) during the implementation phase was commonly emphasized by donor respondents. The respondents reported the partnership between the Bank, DFID, and the government as effective, with regular consultations held through Bank and DFID offices in Beijing [55, 67]. The parties involved had already consented to establish a long-term system to meet the population’s health needs. Therefore, occasional administrative challenges caused by differences in operational cultures were resolved by aligning with decisions made by the government [55]. For example, the Bank accepted the suggestion from a local Chinese technocrat, making sufficient MFA preparation as a condition for activating facility construction in each project county [55]. This suggestion accommodated subnational governments’ preference over facility construction and reluctance to support MFA, facilitating MFA implementation.

“The World Bank, in grasping different aspects of the country’s circumstances and context, relied on local experts and was very pragmatic. Its opinion was not decisive; it instead respected the views of local experts. Once the local situation was well understood, some exploration and pilots would be carried out through the project.” (R15-WB)

The project also adapted to align with the government’s reform agenda, transitioning the project MFA to the national medical assistance program since the government issued its first rural medical assistance policy in 2003 [55, 70, 72]. Donors provided technical support for informing further policy-making through evidence-based research and policy discussions on medical assistance’s financing modalities, procedures, reimbursement standards, and monitoring and evaluation [70], as the following sections indicate.

#### Long-term local expertise cultivation and knowledge generation

The donor respondents highlighted the role of international–domestic expert pairs that promoted long-term knowledge sharing and mentoring. International experts provided expertise on MFA, while the Chinese counterparts sought to enhance their leadership and technical capacities through technical assistance and international exposure to health policy issues. Domestic MFA experts reported positive, lifelong effects of this partnership, which contributed to mindset changes [55, 70]:

“B [an anonymized Bank medical assistance expert] was an inspirational mentor for medical assistance in China because no one knew what medical assistance was before. B was the original mover and shaker of this idea in China and a practitioner. I associate the transformation of my life with B. One winter, we had an all-night conversation, and B changed my entire career—I devoted it to medical assistance.” (R6-WB)

A DFID-funded Core Supervision Team played a vital role in the BHSP’s paired-up approach, ensuring proper supervision by the Bank and DFID. Made up of three international experts, the team saw itself as a bridge and facilitator, rather than as “technical advisors” [67]. It aimed to help all parties understand policy and implementation issues, resolve conflicts, and assist project counties in making informed decisions on suitable health reform paths [55, 67]. However, there was some resistance from the Chinese counterparts, who perceived the role of international experts as lecturers and advisors. This resistance limited the engagement of international experts with local realities and was considered a drawback of the BHSP [67].

Domestic experts, cultivated through the BHSP, were thus entrusted with determining the relevance of specific project ideas to the local context [67]. According to respondents, the government actively involved domestic MFA experts in formulating the national rural medical assistance policy launched in 2003. In 2004, these experts became the country’s expert panels for national medical assistance, supporting the Ministry of Civil Affairs in developing the Guide for Medical Assistance Implementation in rural areas of China [55]. Compared to domestic officials and administrative staff who participated in the BHSP, these experts were more stable in personnel turnovers and had a greater influence on policy-making:

“C [an anonymized domestic MFA expert] and C’s colleagues, who were still in the health economics academia, had the opportunity to be requested by the national policymakers and contribute their insights and expertise in their professional domain, so that they could shape the policy-making process… Influencing policy has been the most critical long-term impact of the BHSP, and that’s how the project served its function for self-sustaining development.” (R16-WB)

Besides, donors also supported generating evidence-based local knowledge. For example, the Ministry of Health and the Ministry of Civil Affairs identified some operational challenges in implementing the national medical assistance policy issued in 2003. The DFID subsequently organized a series of rural health operational studies to address these challenges, involving the domestic MFA experts, during which the relevant government departments took note of the MFA experience [67, 70]. Additionally, the Bank, DFID, and the government consistently encouraged and supported domestic experts to document their project experiences and publish related research articles and books. Donor and subnational government respondents believed that the generated research was the foundation for the domestic MFA experts’ policy advice in national medical assistance and further contributed to the healthcare reform in 2009, as mentioned below.

#### Sustaining project legacies through policy discussions

The Bank, DFID and the government co-organized numerous informal and formal inter-ministerial workshops, seminars, and conferences to promote debate and information-sharing among project participants and various ministries involved in the health sector. These ministries included the Ministry of Civil Affairs, which is responsible for national medical assistance [56, 67]. Particularly, after the issue of Decisions on Strengthening Health Work in Rural Areas in late 2002 and the outbreak of the Severe Acute Respiratory Syndrome epidemic in 2003, the agenda advanced by the BHSP project became part of the central focus of the national health policy debate. The Ministry of Health, therefore, actively worked to accelerate BHSP implementation and organized seminars during project supervision missions to facilitate national policy discussions and disseminate project results [55]. Overall, the respondents perceived these policy discussions as integral to the ongoing efforts to inform China’s health system reforms:

“These lively policy debates provided a platform for discussing various policies. The project then happened to align with some policies being considered by the government, which had already been experimented by the project.” (R3-WB)

Around the project’s conclusion, the government invited the Bank to provide consultation for the country’s new master plan of health system reform, which was subsequently launched by the national government in 2009. This plan served as the basis for improving and institutionalizing the medical assistance policy [56]. The government further invited the Bank to participate in the independent mid-term review of the 2009 healthcare reform in 2012 [73]. The respondents and relevant documents [55, 70, 74] have recognized that the project MFA catalyzed the development of the national rural medical assistance program in China. According to domestic respondents, the national program has evolved significantly since its inception. It almost covers the entire population in poverty across the country [75], indicating its broader coverage and impact compared to the project MFA. Furthermore, it has become more institutionalized and standardized, ensuring its sustainability and effectiveness.

### HIV/AIDS civil society engagement supported by the Global Fund (2010–2013)

The existing literature indicates that the RCC took place in a context where the Fund-supported programs came into a transitional phase for assuming the financial and political responsibilities of the Chinese counterparts on HIV/AIDS civil society engagement [76]. Previous rounds supported by the Fund facilitated convergence between the Chinese and the global HIV/AIDS paradigm. They facilitated the institutionalization of the role of civil society organizations (CSOs)^1^ in a government-dominated socio-cultural context [54, 77] and empowered emerging CSOs and marginalized and HIV-positive individuals to serve their communities and voice their needs [57, 78]. Moreover, amid rapid private wealth accumulation and social inequity challenges, the government gradually recognized the CSOs’ role in reaching marginalized, HIV-affected populations for health services and embraced international cooperation [79, 80]. This resulted in domestic HIV/AIDS CSOs collaborating with transnational actors and adopting global norms, as highlighted by respondents and existing literature [80].

During the RCC, the Fund sought to solidify its legacies by strengthening civil societies, notably being the most supportive external donor in advancing community-based approaches in China’s HIV/AIDS response [57]. Particularly, it supported a CBO program within RCC from 2012. However, the government became increasingly concerned about political infiltration through externally-endorsed CSOs and prioritized regime security during this period [77, 80, 81]. Respondents also observed that CSOs in China were still in the early stages of development. How the donor and recipient addressed the sustainability challenges of HIV/AIDS civil society engagement thus became a significant concern in this case study.

#### Inadequate dialogues

**Fig. 2:**
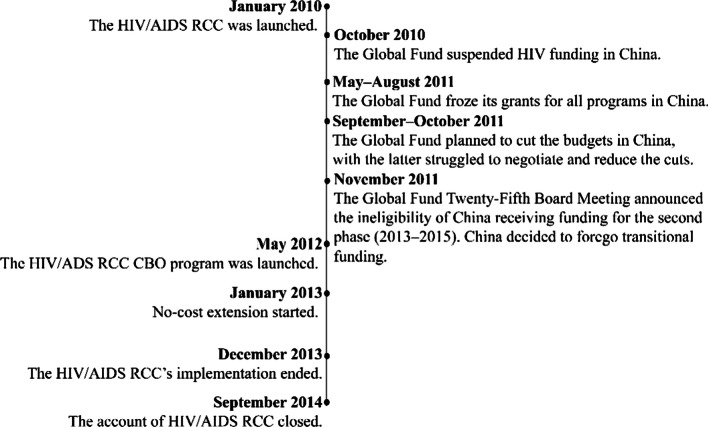
Timeline of the RCC and the unilateral events (Abbreviation: *CBO* Community-based organization; *RCC* Rolling Continuation Channel). Source: [78, 84, 85]

Without an in-country office, the Fund communicated with the Chinese counterparts through the Country Coordinating Mechanism (CCM)^2^ and CCM working groups, particularly during the RCC, to engage in dialogues regarding civil society involvement [78]. Unfortunately, this distance-based approach made the donor–recipient dialogues inadequate, leading to misunderstandings, mistrust, and unilateral action (Fig. 2) on both sides.

The misunderstandings initially arose regarding the concept of ‘consolidation’, which contributed to the complexity of management during the transition period of the Fund-supported programs. Specifically, one vision of the RCC was to consolidate efforts and resources in the previous rounds of the Fund for a smooth transition [78]. The government interpreted this ‘consolidation’ as integrating international resources into the national budget to support government policies [86, 87]. On the other hand, the Fund understood consolidation as employing stricter financial and accountability standards to mitigate risks in program performance before the Fund’s exit, reflecting its nature as a performance-based initiative accountable to its funders [57]. The national government and expert respondents perceived Fund managers as presuming inadequate government attention to corruption and inefficiency in communications with their Chinese counterparts. Consequently, the Fund maintained a separate system for stricter program management, auditing, and reporting, increasing the burden of micro-management [78, 88].

The stricter management imposed by the Fund, combined with the ambiguity surrounding the definition of CSOs and related standards, resulted in a freeze of HIV/AIDS grants [85]. CSO respondents noted that the national government favored government-affiliated organizations, including government-organized non-governmental organizations (GONGOs) and community health centers, contrary to the Fund’s emphasis on community-based organizations (CBOs)^3^ [86]. Due to this ambiguity, several leaders from community-based organizations accused the government of mismanaging the RCC project, complaining to the Fund that government-affiliated organizations received most of the funds allocated to CSOs. The result was a series of “unpleasant disputes” between the Fund and the government, closed meetings between the Fund and community representatives, and the Fund’s unilateral administrative letter reporting mismanagement to the Chinese CDC, the Principal Recipient of the RCC [89, 90]. Although subsequent reviews by the Fund and independent evaluations found no significant mismanagement and fund misuse, they highlighted the Principal Recipient’s compliance problems with CBO selection [57, 78, 85, 89–92]. As a result, the Fund temporarily suspended and then froze the grants for HIV/AIDS [93]. The government ultimately agreed to ensure community engagement and proper financial management for the grant resumption [57, 77, 90]. This event, however, led to an interruption in the implementation of CBO projects [88]. Consequently, the government lost its reputation, and there was increased mistrust towards the Fund, as reported by domestic respondents.

“Accusing the Global Fund’s project—(the Ministry of Health managed the project)—meant accusing the government, which was humiliating to the government. The government was very uncomfortable that the money was monitored by the Global Fund, who did not provide much money. It [the money freeze] also caused a stir in the international public opinion [towards China], so it was right to close it [the RCC project].” (R26-GF)

As the pressures from Fund’s funders to exit China intensified due to the country’s graduation to an upper-middle-income status [77, 94], the bilateral relationship between China and the Fund further deteriorated [95]. The Fund’s Board made the exit decision without prior consultation with the Principal Recipient or the CCM, and announced this decision in November 2011, stating that China was ineligible for renewals of Phase II grants from 2013 to 2015 [96]. In response to this sudden decision, the CCM HIV/AIDS Working Group and the Fund’s Office of the Inspector General identified risks and proposed mitigation solutions related to CBO funding and service quality [91]. More bilateral discussions at a higher level and frequent visits from the Fund to China also took place [78].

However, due to the requests from Fund funders to reduce funding commitments to China and minimize operational risks, communication between the Fund and the government mainly focused on operational mechanics, budget cuts, and the country’s compliance with Fund agreements and financial standards [78]. On the other hand, the government lost interest in dialoguing with the Fund regarding transitional plans [78]. While the Fund supported transitional investigations for civil society engagement [97], the government unilaterally decided to request no-cost extensions and declined the Fund’s offer of transitional funding [57, 78]. The transition planning was then internally undertaken by domestic actors [98].

These unilateral actions had negative consequences for HIV/AIDS CSOs, leading to a policy vacuum and dependence of CSOs on the government [99, 100]. A great proportion of HIV/AIDS CSOs were forced to close or leave the field, as observed by CSO respondents. Moreover, as the Fund and other external donors for HIV/AIDS exited, international–domestic communication has drastically decreased. China’s CSOs were more isolated from transnational HIV/AIDS networks in the global arena than before, described by a CSO respondent as “closing eyes for looking at the globe” (*R36-GF*). These factors collectively contributed to a challenging environment for CSOs in China and hindered their ability to address the HIV/AIDS epidemic effectively [99, 100].

#### The Global Fund’s compromise with a dominant government

In consolidating the previous rounds supported by the Fund, the government became dominant in adopting the community-based approach in the RCC implementation [57, 94]. Pressured by a performance-based funding mechanism, however, the Fund chose not to confront the government when concerns about CSO sustainability arose, as reported by CBO respondents. For example, despite the potential benefits of the RCC’s decentralization [54], resistance and mistrust from subnational governments constrained civil society expansion in China [54, 57, 89, 101]. The Fund was aware of these challenges but chose to align with the national decentralization policy in HIV/AIDS response [54, 102], leaving behind the potential systematic risks that undermined CSOs’ sustainability to complete its programs in China [57].

The RCC’s alignment with the HIV/AIDS response system led by the Chinese CDC further resulted in CSOs being relegated to a supplementary role in service delivery for marginalized populations, as revealed by respondents. The CDCs and GONGOs were criticized for occupying important positions within RCC governance and being eager to spend money, while CBOs were encouraged to compete for targets set by the CDCs [99]. In this vein, the Fund remained consent with these “simplified, tangible tasks” (*R37-GF*). This compromise resulted in increasing bureaucratization and medicalization among CSOs and subsequently constrained CBOs’ ability to innovate and improve interventions for the beneficiaries [99]:

“The leadership of this organization [an anonymized GONGO] diluted the vision of civil society engagement. It excused that it could not achieve this vision, and then continuously compromised… From the national to the provincial to municipal levels, the organization charged management fees… Even if a CBO project could not be implemented, they were anxious to use up this money. The CBOs were thus reluctant to implement the project… so, in this case, the CBO projects have become a ‘seller’s market’.”(R37-GF)

#### True civil society engagement?

While the compromise with government dominance limited the Fund’s ability to promote broad-based human rights activism [55, 95], the Fund added civil society engagement as a condition in the RCC. As GONGO and government respondents reported, this conditionality left the impression of the Fund as “being politicalized” and a “back-seat driver” (*R23-GF*). For example, it requested a certain funding proportion for the communities or a certain number of centers for female sexual workers, without adequate consideration of the communities’ insufficient human resources and capacities. This conditionality led to the emergence of CSOs perceived as “fake” by the respondents and some CSO leaders,^4^ bringing ineffectiveness and counterproductive competition for limited resources, thus resulting in fragmentation within the CSO network under the CDC-led HIV/AIDS response system [57, 99].

As observed by the CSO respondents, the governance of civil society engagement in the RCC might have brought counteractive effects for post-transition governance. “CBO frauds in the election process” of CCM (*R31-GF*) for “the Western democracy” as well as “interference by the UNAIDS” (*R41-GF*) have set off the government’s security concerns [57, 99]. As a consequence, the post-transition dissolution of the multi-stakeholder governance limited CSO representation to very few “well-performing”, elitist CSOs (*R34-GF*), and a focus on cross-departmental efforts led by the State Council AIDS Working Committee Office, hindering an open, transparent, multi-stakeholder governance mechanism [57, 99].

As a result, many CBO respondents observed that the RCC failed to fully sustain genuine civil society engagement in China’s HIV/AIDS response after the Fund’s exit. The post-transition programs, CAFNGO and subnational government contracting programs of services from CSOs, are said by respondents to fit Chinese contexts and have stricter, improved management. However, CSOs have largely become “passive helpers” *(R27/37/ 41/44-GF*), constrained by government requests. Subnational support for CSO development, on the other hand, varies depending on the local government’s stances^5^ [77, 81]. While a few provinces provide continuous, inclusive financial, political, and legislative support for CSO development, most government-supported programs focus explicitly on target-oriented testing and treatments, less mentioning advocacy, informational exchanges, and mental and social network support services [104, 105]. They also introduce market principles, favoring larger, registered CSOs trusted by the government and leading to specialization and medicalization of CSOs. Therefore, many unregistered CBOs and community groups, reliant on government funding and supervised by the registered CSOs or local CDCs, have deviated from their rights-based mission [77, 81, 86, 99, 100]. The respondents used “great waves sweeping away sands” (*da lang tao sha*) to describe the process of sifting “incapable CSOs” (*R26/29/35*-*GF*).

“In the past, international donors gave the money directly to CSOs, so the country’s CSOs depended on these donors. But now the country has stopped this. There is no need to listen to the donors. [The government] would give them the requests and methods for completing the tasks, which match the targets and indicator system set by the CDCs. You cannot say this is wrong, but it is more of a performance-based and corporate way of management… CSOs cannot think widely and act innovatively because now the government would tell you the designated move.” (R27-GF)

## Discussion

Based on the collected data, the selected cases exhibit diverse donor–recipient dynamics and outcomes regarding transnational policy diffusion. This section identifies three interconnected and reemergent aspects of donor–recipient dynamics observed across the cases and explores alternative explanations, while engaging with existing literature that presents similar or divergent findings. The implications and limitations of this study are also discussed.

### Reemergent aspects of donor–recipient dynamics in the case study

**Fig. 3:**
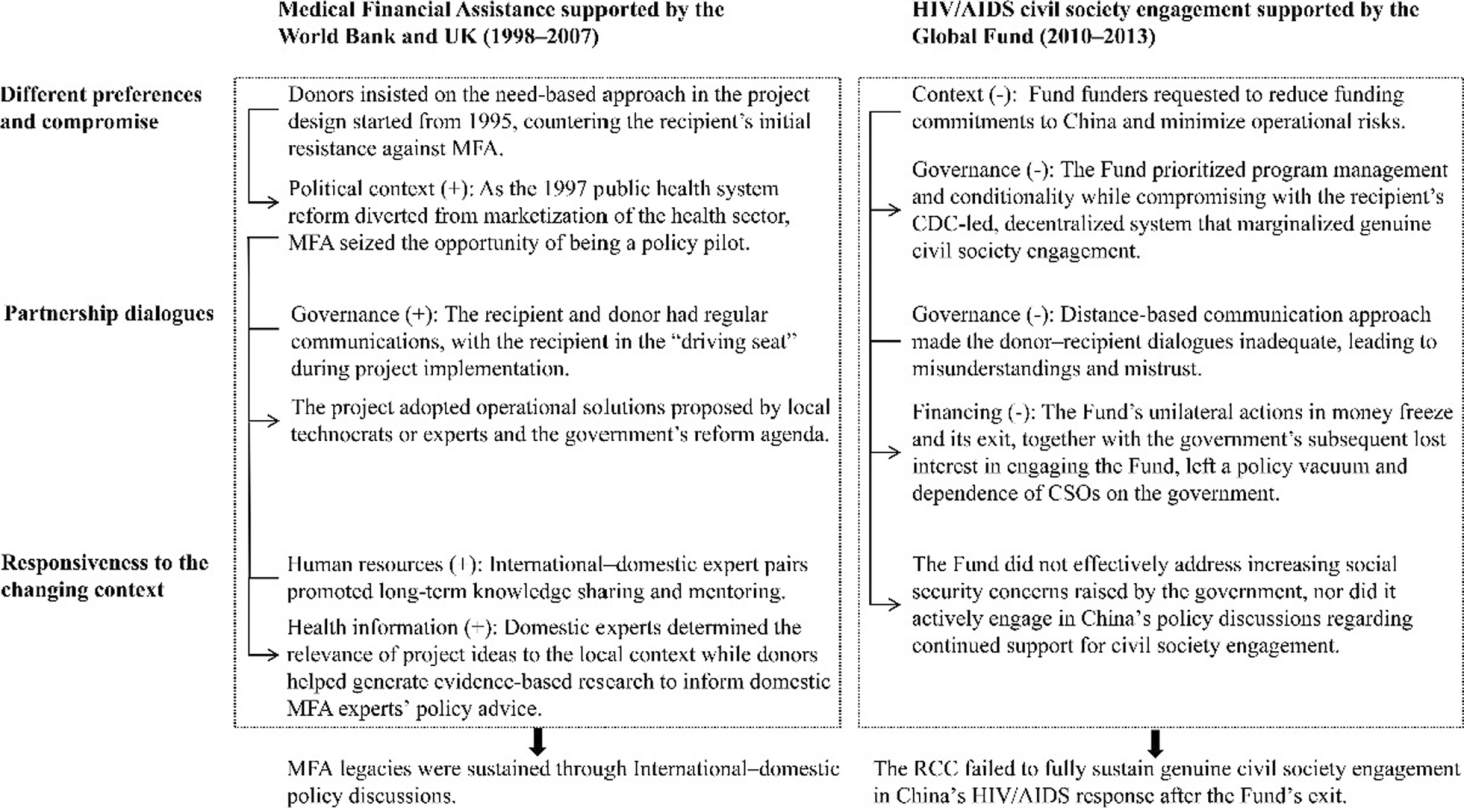
Key findings from the case study

Figure 3 provides a summary of the main findings from the cross-case study, which are categorized into three interrelated and recurring aspects: (1) different preferences and compromise, (2) partnership dialogues, and (3) responsiveness to a changing context. These findings primarily focus on the roles and actions of the involved actors, highlighting the key processes. The figure also showcases relevant enablers (indicated by “+”) and barriers (indicated by “−”) identified from the “content” and “context” within the policy triangle framework.

#### Different preferences and compromise

Both cases highlight the importance of project and policy ownership of the recipient [106]. However, in this case study, there seems a stark contrast between the Bank and DFID’s long-term commitment to addressing local needs, and the Fund’s tendency to prioritize program management for situational convenience, which has also been observed in other global health initiatives [24, 30]. The donors’ principled preference and compromise, together with the recipient’s pragmatism and strong ownership for long-term health needs, allowed the BHSP to accumulate managerial and technical experience in medical assistance and seize the policy window. On the other hand, the Fund compromised with the CDC-led, decentralized system that marginalized genuine civil society engagement. Its preference instead turned to strict management, bypassing transitional planning. This study thus demonstrates that strategic and selective preferences and compromise by the donor and recipient can reach a balance between donor-advocated global health norms and practices and recipient ownership to ensure post-DAH sustainability, aligning with some existing literature [11, 57].

#### Partnership dialogues

The concept of “partnership” represents a shift from donor paternalism towards equal collaboration and recipient independence in development assistance, reflected in how donor–recipient dialogues are constructed [29, 34]. In the case of MFA in BHSP, the donor–recipient dialogues were characterized by on-site mutual learning and understanding, fostering a long-term and strong partnership that informed the country’s health system reforms. On the other hand, insufficient dialogues between the Fund and the Chinese counterparts and the subsequent exclusion of the Fund in the transitional planning of the HIV/AIDS RCC might have left the fate of nascent civil society engagement uncertain in a government-dominant context after the Fund’s exit. This study thus highlights that donor–recipient dynamics could evolve to a point where dialogues become collaborative, based on consensus, trust, and voluntary participation in domestic policy formulation [4, 7, 13, 28, 45]. These collaborative dialogues could positively impact the recipient’s perception of DAH benefits for policy formulation and health outcomes and the donor’s understanding of domestic policy actors’ strategic objectives, policy preferences, and tools [107].

#### Responsiveness to the changing context

In the case of BHSP, donors’ technical assistance played a crucial role in empowering local project participants to localize MFA. This included building capacity for local expertise and knowledge generation and facilitating domestic policy discussions, echoing other case studies [22, 42, 107]. Thus, the project MFA aligned its design with the 1997 public health reform, informed the formulation of national medical assistance strategies in 2003, and provided valuable lessons for the 2009 healthcare reform. In contrast, the Fund demonstrated a relatively passive approach in responding to the changing context in the selected case. According to the collected data, it did not effectively address increasing social security concerns raised by the government, nor did it actively engage in China’s policy discussions regarding continued support for civil society engagement. Rather, in response to the pressure from Fund donors, it suddenly ceased funding for China, hampering post-DAH sustainability. Based on this comparison, this study thus highlights proper, proactive responsiveness to the changing context via building local expertise, generating local knowledge, and engaging in domestic policy discussions.

### Alternative explanations

While the existing literature on policy diffusion has identified potential alternative explanations on other factors that might influence transnational policy diffusion through DAH, the two cases exhibit similarities in these factors, suggesting that these may not be the key determinants of the differences in policy diffusion outcomes between the cases. One important factor scholars highlighted is the critical juncture for policy reforms [108]. However, while there was a leadership change and the outbreak of SARS in 2002–2003, which favored prioritizing medical assistance [55, 67], the government also demonstrated a strong political commitment to address concerns about the financial gap in HIV/AIDS CSOs, particularly in response to the Fund’s exit in 2012 [81]. Aligning with other scholars’ appeal for going beyond the context in identifying causal patterns [42], we thus highlight the importance of the donor and recipient’s detection of and responsiveness to the context. Another alternative factor is the political sensitiveness of the DAH-supported intervention [34], specifically its degree of undermining the ideology, values, and security of the ruling regime [38, 40, 109]. However, in the BHSP, as the government embraced developmentalism to maintain regime legitimacy [110], the government once doubted the effectiveness of MFA in boosting development. In the RCC, the government also adopted a securitization approach to civil society engagement to keep regime security [77]. Measuring and comparing political sensitiveness between cases was thus challenging.

Other potential alternative factors in the policy triangle framework also show similarities across the two cases, such as governance (e.g., project management structure and leadership development), monitoring and evaluation (e.g., project performance), human resources (e.g., capacity building), and the recipient’s general ownership independent from donors. Therefore, we reclaim that donor–recipient dynamic is a significant factor in transnational policy diffusion through DAH.

### Implications and other consideration

While consistent with other qualitative case studies [31, 46, 52], the study identifies three interrelated aspects of donor–recipient dynamics in DAH that influence domestic health policy formulation, with the following implications.

First, donors should strategically balance their preferences with the recipient’s ownership, acknowledging potential rejections and seeking local adaptations without compromising the core values of the interventions. Both parties’ long-term commitment to addressing local needs is essential. Additionally, the recipient ownership that is merely aimed to hand over the DAH-supported interventions to the domestic counterparts without comprehensively considering the local context, should be critically evaluated [29, 111].

Second, donor–recipient partnership dialogues are crucial for mutual understanding and learning. These dialogues can be strengthened through formal negotiations, informal communication, and personal relationship building.

Third, proactive efforts are needed to identify and react to contextual enablers and barriers. Collaborative, in-depth international–domestic expert partnerships should be built to conduct country sector studies, engage in evidence-based policy advocacy, and design risk mitigation plans for leveraging contextual enablers and overcoming barriers [9, 30].

Our case study, despite in the setting of China, fits into circumstances where DAH projects can serve as pilots for domestic policy formulation, which aligns with studies that demonstrated the autonomy of countries like Vietnam, South Korea, India, Ethiopia, and Rwanda in adapting external policy ideas and ensuring post-DAH sustainability [22, 29, 34, 38, 40]. Moreover, China’s recent graduation from DAH presents an opportunity for China, as an emerging donor, and other donors to continue or reestablish their development partnerships and shape their DAH transition strategies. Therefore, while China’s DAH transition experiences may not be fully transferred in other settings, they can offer practical lessons for health development cooperation in the Global South.

It is important to note that this study does not assume a clear divergence of policy goals, ideas, and tools between the donor and recipient. Thus, this study’s focus is not on the extent to which donor-imported policies were adopted. Also, as various domestic and international factors influenced the outcomes of the focused policies, the study does not dichotomously categorize policy diffusions in the two case projects as either successful or failed, or dictate what the two focused policies should be like in China. Finally, while the study calls for vitalizing donor–recipient dynamics in DAH, it does not endorse or legitimize arbitrary external influence that undermines the host country’s sovereignty and ownership.

### Limitations

Despite our best efforts, this study has inherent limitations due to constraints in research capacity, time, and resources. Firstly, as a case study focusing on two cases within a specific timeframe in a single country, it can only provide a contextualized perspective, without capturing the full complexity and diversity of the research topic.

Secondly, there were disparities between the two targeted interventions. These disparities affected our ability to contact interviewees, with a more positive response from the BHSP informants than the RCC. In addition, other donors, such as the Bill & Melinda Gates Foundation, also supported HIV/AIDS civil society engagement despite on a smaller scale. This study could not exclude their influence. However, we prioritize the richness derived from diverse donors and interventions and the depth of the case study over strict control of variables.

Thirdly, this retrospective study introduces the possibility of recall bias. Due to personnel movement in the past decade, government respondents were primarily limited to those in the health and social security sector. While we faced difficulties contacting community or beneficiary stakeholders engaged in the project and policy formulation process, the existing respondents, documents, and literature also provided limited information on community and beneficiary participation in transitioning the two DAH-supported interventions into the local health system. Data saturation has also not been reached for donor stakeholders of the RCC case. Nevertheless, we have tried to triangulate across data sources, and our interviews have covered diverse perspectives to the best of our ability.

## Conclusions

This study argues that the donor–recipient dynamic is crucial in transitioning DAH-supported interventions into domestic health policy. Based on evidence from two health interventions supported by the World Bank–UK and the Global Fund, the study emphasizes the significance of balancing donor priorities with recipient ownership, partnership dialogues, and proactive responsiveness to the changing context in achieving effective domestic health policy formulation and post-DAH sustainability. Additionally, we recommend conducting program evaluations on DAH transition for better routine data collection and closer monitoring of changes. Further empirical research on this topic in China and other settings is also needed to enhance the understanding of the subject matter.

### Supplementary Information

**Additional File 1.** A scoping review on the Basic Health Services Project supported by World Bank and UK and HIV/AIDS rolling continuation channel supported by Global Fund in China. **2.** Major project and policy documents and multimedia. **3.** Each respondent’s profile. **4.** Examples of the interview guide.

## Data Availability

Upon the request of IRB, interview transcripts used for the analysis are confidential, as they could be sources to identify the participants. The detailed results of the review of related literature and documents (see search strategy in Additional file 1) are available from the authors after deidentification and anonymization, upon reasonable request. Additional file 1 lists policy and project documents as data sources of this study.

## Abbreviations

BHSP: Basic Health Services Project
CAFNGO: China AIDS Fund for Non-Governmental Organizations
CBO: Community-based organization
CCM: Country coordination mechanism
CDC: Center for diseases control and prevention
CSO: Civil society organization
DAH: Development assistance for health
DFID: (UK) Department for International Development
GONGO: Government-organized non-governmental organization
MFA: Medical Financial Assistance Scheme
RCC: Rolling continuation channel

## Footnotes

1 In this study, ‘civil society organization’ and ‘non-government organization’ are used interchangeably and in accordance with the government’s official documents. Civil society organizations include community-based organizations, government-organized non-governmental organizations, and other civil society organizations.

2 The CCM functioned through multi-stakeholder mechanisms for tackling with HIV/AIDS, malaria, and tuberculosis. Data from 2006 to 2012 showed that two among the 22 seats in CCM were reserved for one elected representative of CBOs and one from the community of people living with HIV/AIDS, malaria or tuberculosis, with two representatives of HIV/AIDS CBOs and people living with HIV/AIDS both being members within this period [82, 83]. Specifically, five seats were for the conveners, including Chair, Vice Chair, Chair of AIDS Working Group, Chair of TB Working Group, Chair of Malaria Working Group. Five seats were for the government sector, with one for the Ministry of Health and four for other central government’s agencies. Six seats were for the non-government sector, including the Chinese Center for Disease Control and Prevention, professional or educational institutions, mass or social organizations, CBO and other NGO, and International NGO. One seat was for a patient representative, and another for a private or state-owned enterprise. The rest four seats were for international organizations, including those multilateral and bilateral [83].

3 “Community-based organizations” refer to organizations formed by people from the same community (e.g., men who have sex with men [MSM], injection drug users [IDUs], commercial sexual workers [CSWs], people living with HIV [PLHIV], etc.).

4 For example, according to Liu [87], a leader of the National AIDS Information Network (CHAIN) categorized China’s CSOs during the Fund’s support as below: (1) spontaneous organizations that already existed and had been undertaking community services and advocacy work before the Fund entered China; (2) project-induced organizations that born of the Fund’s support, established for completing work on the Fund projects; (3) parasitic organizations, or “fake organizations”, with staff belong either to the CDC or are part of already-existing intervention teams.

5 For example, Yunnan and Sichuan province provides continuous financial, political, and legislative support for CSO development [81, 103]. However, in other areas, respondents reported political sensitiveness of sexual minorities and commercial sexual workers’ rights [81] and the local government usually circumvent these issues, thus refusing genuine continuous support for CBOs.
