# Supplementary material for: Development assistance, donor–recipient dynamic, and domestic policy: a case study of two health interventions supported by World Bank–UK and Global Fund in China
Source: Glob Health Res Policy. 2024 Feb 4;9:7. doi: 10.1186/s41256-024-00344-3 (PMC10838425; doi:10.1186/s41256-024-00344-3)
Supplement: Supplementary file 1 — Additional File 1. A scoping review on the Basic Health Services Project supported by World Bank and UK and HIV/AIDS rolling continuation channel supported by Global Fund in China. 2. Major project and policy documents and multimedia. 3. Each respondent’s profile. 4. Examples of the interview guide. [file 41256_2024_344_MOESM1_ESM.docx]

**Additional File 1. A scoping review on the Basic Health Services Project supported by World Bank and UK and HIV/AIDS rolling continuation channel supported by Global Fund in China**

We conducted a scoping review of related published academic literature as well as policy and project report/documents that analyzed the design, implementation, and influence of medical financial assistance of the Basic Health Services Project (BHSP) as well as civil society engagement of HIV/AIDS rolling continuation channel (RCC) in China. The objectives of this review were (1) to identify existing sources documenting these two projects, (2) extract data for the research project ‘Sustaining effective coverage in the context of transition from external assistance – Lessons from China’, and (3) support analysis for sub-themes of this broad research project, such as ‘development assistance, donor–recipient dynamic, and domestic policy’ in this study. As we aimed to conduct case study on these two projects and found few studies summarizing literature and documents on these projects, we believed a scoping review necessary for investigating the two projects comprehensively.

We combined terms and phrases related to World Bank, DFID, and the BHSP, as well as Global Fund and HIV/AIDS on Embase, MEDLINE, and China National Knowledge Integrated Database (see search strategy below). We also conducted additional searches on Google, Google Scholar, World Bank Open Knowledge Repository and eLibrary, the Overseas Development Institute, and the official websites of the World Bank, the UK government, the Chinese Centers For Disease Control And Prevention, and the Global Fund. We also identified additional studies or documents by reviewing the reference lists of related published literature and secured them through online searches. The search limited the publication date from 1995 to 2020. Some studies or documents were obtained through personal correspondence, which might be published after 2020. Throughout the data collection process, the relevant policy documents were identified as the project documents, related literature, and key informants indicated some of them.

We included all studies if they analyzed each specific project examined or are relevant to the general topics that could be helpful to our understanding of the transition context (e.g., analyzing the trend and progress of HIV/AIDS civil society engagement in China); we excluded studies if they did not mention the specific projects or have a very limited analysis of them that do not contribute to our understanding of the project design, implementation and transition. We conducted two rounds of screening, first title and abstract and then full-text screening, and all screening was conducted by one team member whereas uncertain ones were discussed with another team member. A total of 8300 citations were screened.

Finally, we included 64 studies and documents for the BHSP case and 65 for the RCC case for this study. Data charting followed the policy triangle framework while data synthesis has been scored in MAXQDA, triangulated with data from our interviews. As this study’s focus is not the scoping review, and a long report of the review process will distract the readers’ attention, we highlight a list of major literature and documents in Additional file 2. In the same vein, as this scoping review aimed not to report the sources per se, but to extract secondary data for the qualitative research mentioned above, ‘results’ and ‘discussion’ in the PRISMA for scoping reviews are basically not applicable here.

Search date: February 4, 2022

Embase and MEDLINE through OVID

| # | Searches | Results |
| --- | --- | --- |
| 1 | ("World Bank" or "WB" or "IDA" or "International Development Assistance" or "DFID" or "Department for International Development" or "China").ab,kw,ti. | 554507 |
| 2 | ("Basic Health Services Project" or "Health VIII" or "H8SP" or "Health Eight Support Project" or "H8/SP" or "Qinba Health program").af. | 42 |
| 3 | 1 and 2 | 5 |
| 4 | remove duplicates from 3 | 3 |

| # | searches | results |
| --- | --- | --- |
| 1 | china.kw,ti,ab. | 504973 |
| 2 | ("GFATM" or "Global Fund").ab,kw,ti. | 1769 |
| 3 | ("AIDS" or "HIV").ab,kw,ti. | 940237 |
| 4 | 1 and 2 and 3 | 41 |
| 5 | remove duplicates from 4 | 24 |

China National Knowledge Integrated Database (“中国知网”)

Language: Chinese

Searches: ( ( ( ( 旧版主题= '世行'+'世界银行'+'世界银行贷款' ) AND ( 旧版主题= '卫八'+'卫生Ⅷ'+'卫生Ⅷ项目'+'基本卫生服务项目'+'秦巴卫生项目'+'卫生Ⅷ支持性项目' ) ) OR ( 关键词='卫生Ⅷ项目' ) ) NOT ( 文献来源%'年鉴' or 中文刊名%'年鉴' ) ) AND ( 发表时间 Between('1995-01-01','2020-12-31') )

Result: 1315

Searches: ( ( 旧版主题= '全球基金'*'艾滋病' ) OR ( 主题= '全球基金'*'艾滋病' or 题名= '全球基金'*'艾滋病' ) NOT ( 文献来源%'年鉴' or 中文刊名%'年鉴' ) ) AND ( 发表时间 Between('2003-01-01','2020-12-31') )；检索范围：中文文献。

Result: 493

Google and/or Google Scholar

| Search strategy | | Results | |
| --- | --- | --- | --- |
|  |  | Google | Google Scholar |
| Searches (English) | (“World Bank” OR “WB” OR “IDA” OR “International Development Assistance” OR DFID OR “Department for International Development” OR China) AND (“Basic Health Services Project” OR “Health VIII” OR H8SP OR “Health Eight Support Project” OR H8/SP OR “Qinba Health program”) | 83 | 54 |
| Searches (Chinese) | (世行 OR 世界银行 OR 世界银行贷款) AND (卫八 OR 卫生Ⅷ OR 基本卫生服务项目 OR 秦巴卫生项目) | 123 | 836 |
| Filtered year | 1995-2020 |  | |
| De-customized | Yes |  |  |

| Search strategy | | Google |
| --- | --- | --- |
| Search string (English) | China AND ("Global fund" OR GFATM) AND (HIV OR AIDS) | 116 |
| Search string (Chinese) | “全球基金” AND（“艾滋病” OR AIDS OR HIV） | 119 |
| Search string (Chinese) | 全球基金艾滋病项目 | 115 |
| Filtered year | 2003-2020 |  |
| De-customized | Yes |  |

World Bank websites

| Website | Searches | Results |
| --- | --- | --- |
| World Bank Open Knowledge Repository | China AND (“Basic Health Services Project” OR “Health VIII” OR H8SP OR “Health Eight Support Project” OR H8/SP OR “Qinba Health program”) | 1111 |
| World Bank eLibrary | China basic health services | 629 |

Overseas Development Institute

| Searches | Results |
| --- | --- |
| basic health services project China | 7 |
| Health VIII China | 1 |
| Health Eight Support Project China | 113 |
| H8SP, H8/SP, Qinba health program | 0 |

Official websites:

1. World Bank Projects & Operations

https://projects.worldbank.org/en/projects-operations/project-detail/P003566

1. UK government

https://www.gov.uk/search/all?keywords=basic+health+services+project+China&level_one_taxon=37d0fa26-abed-4c74-8835-b3b51ae1c8b2&level_two_taxon=9fb30a53-70fb-4f1c-878b-0064b202d1ba&order=relevance&page=1

1. Chinese Centers For Disease Control And Prevention

http://ncaids.chinacdc.cn/2018zlxz/201401/W020140129341403960268.pdf

1. Global Fund
   1. https://data.theglobalfund.org/grant/CHN-304-G03-H/2/documents
   2. https://www.theglobalfund.org/en/oig/reports/
   3. https://www.theglobalfund.org/en/board/decisions/

**Preferred Reporting Items for Systematic reviews and Meta-Analyses extension for Scoping Reviews (PRISMA-ScR) Checklist**

| **SECTION** | **ITEM** | **PRISMA-ScR CHECKLIST ITEM** | **REPORTED ON PAGE #** |
| --- | --- | --- | --- |
| **TITLE** | | | |
| Title | 1 | Identify the report as a scoping review. | Page 1, Additional file 1 |
| **ABSTRACT** | | | |
| Structured summary | 2 | Provide a structured summary that includes (as applicable): background, objectives, eligibility criteria, sources of evidence, charting methods, results, and conclusions that relate to the review questions and objectives. | Page 1, Additional file 1 |
| **INTRODUCTION** | | | |
| Rationale | 3 | Describe the rationale for the review in the context of what is already known. Explain why the review questions/objectives lend themselves to a scoping review approach. | Page 1, Additional file 1 |
| Objectives | 4 | Provide an explicit statement of the questions and objectives being addressed with reference to their key elements (e.g., population or participants, concepts, and context) or other relevant key elements used to conceptualize the review questions and/or objectives. | Page 1, Additional file 1 |
| **METHODS** | | | |
| Protocol and registration | 5 | Indicate whether a review protocol exists; state if and where it can be accessed (e.g., a Web address); and if available, provide registration information, including the registration number. | No, as the scoping review is instrumental to the qualitative case study. |
| Eligibility criteria | 6 | Specify characteristics of the sources of evidence used as eligibility criteria (e.g., years considered, language, and publication status), and provide a rationale. | Page 1, Additional file 1 |
| Information sources* | 7 | Describe all information sources in the search (e.g., databases with dates of coverage and contact with authors to identify additional sources), as well as the date the most recent search was executed. | Page 1, Additional file 1 |
| Search | 8 | Present the full electronic search strategy for at least 1 database, including any limits used, such that it could be repeated. | Page 1, Additional file 1 |
| Selection of sources of evidence† | 9 | State the process for selecting sources of evidence (i.e., screening and eligibility) included in the scoping review. | Page 2-4, Additional file 1 |
| Data charting process‡ | 10 | Describe the methods of charting data from the included sources of evidence (e.g., calibrated forms or forms that have been tested by the team before their use, and whether data charting was done independently or in duplicate) and any processes for obtaining and confirming data from investigators. | Page 1, Additional file 1 |
| Data items | 11 | List and define all variables for which data were sought and any assumptions and simplifications made. | Figure 1, Page 9, manuscript |
| Critical appraisal of individual sources of evidence§ | 12 | If done, provide a rationale for conducting a critical appraisal of included sources of evidence; describe the methods used and how this information was used in any data synthesis (if appropriate). | Not applicable |
| Synthesis of results | 13 | Describe the methods of handling and summarizing the data that were charted. | Page 2, Additional file 1 |
| **RESULTS** | | | |
| Selection of sources of evidence | 14 | Give numbers of sources of evidence screened, assessed for eligibility, and included in the review, with reasons for exclusions at each stage, ideally using a flow diagram. | Page 1, Additional file 1 |
| Characteristics of sources of evidence | 15 | For each source of evidence, present characteristics for which data were charted and provide the citations. | Not applicable |
| Critical appraisal within sources of evidence | 16 | If done, present data on critical appraisal of included sources of evidence (see item 12). | Not applicable |
| Results of individual sources of evidence | 17 | For each included source of evidence, present the relevant data that were charted that relate to the review questions and objectives. | Not applicable |
| Synthesis of results | 18 | Summarize and/or present the charting results as they relate to the review questions and objectives. | Not applicable |
| **DISCUSSION** | | | |
| Summary of evidence | 19 | Summarize the main results (including an overview of concepts, themes, and types of evidence available), link to the review questions and objectives, and consider the relevance to key groups. | Not applicable |
| Limitations | 20 | Discuss the limitations of the scoping review process. | Page 2, Additional file 1 |
| Conclusions | 21 | Provide a general interpretation of the results with respect to the review questions and objectives, as well as potential implications and/or next steps. | Not applicable |
| **FUNDING** | | | |
| Funding | 22 | Describe sources of funding for the included sources of evidence, as well as sources of funding for the scoping review. Describe the role of the funders of the scoping review. | Page 43, manuscript |

JBI = Joanna Briggs Institute; PRISMA-ScR = Preferred Reporting Items for Systematic reviews and Meta-Analyses extension for Scoping Reviews.

* Where *sources of evidence* (see second footnote) are compiled from, such as bibliographic databases, social media platforms, and Web sites.

† A more inclusive/heterogeneous term used to account for the different types of evidence or data sources (e.g., quantitative and/or qualitative research, expert opinion, and policy documents) that may be eligible in a scoping review as opposed to only studies. This is not to be confused with *information sources* (see first footnote).

‡ The frameworks by Arksey and O’Malley (6) and Levac and colleagues (7) and the JBI guidance (4, 5) refer to the process of data extraction in a scoping review as data charting*.*

§ The process of systematically examining research evidence to assess its validity, results, and relevance before using it to inform a decision. This term is used for items 12 and 19 instead of "risk of bias" (which is more applicable to systematic reviews of interventions) to include and acknowledge the various sources of evidence that may be used in a scoping review (e.g., quantitative and/or qualitative research, expert opinion, and policy document).

*From:* Tricco AC, Lillie E, Zarin W, O'Brien KK, Colquhoun H, Levac D, et al. PRISMA Extension for Scoping Reviews (PRISMAScR): Checklist and Explanation. Ann Intern Med. 2018;169:467–473. [doi: 10.7326/M18-0850](http://annals.org/aim/fullarticle/2700389/prisma-extension-scoping-reviews-prisma-scr-checklist-explanation).

**Additional File 2.** **Major project and policy documents and multimedia**

*Basic Health Services Project*

Project documents

1. World Bank Group. Project Appraisal Document on a proposed credit of SDR 63.0 million for the People’s Republic of China for a Basic Health Services Project [Internet]. Washington, D.C: World Bank Group; 1998 Apr. (World Development Sources). Report No.: 17403. Available from: http://documents.worldbank.org/curated/en/406281468769778128/China-Basic-Health-Services-Project
2. World Bank Group. Implementation Completion and Results Report on a credit of SDR 63.0 million for the People’s Republic of China for a Basic Health Services Project [Internet]. Washington, D.C: World Bank Group; 2008. Report No.: ICR512. Available from: http://documents.worldbank.org/curated/en/293281468028733713/China-Basic-Health-Services-Project
3. Liu Y, Liu G, Liu M, Xu L, editors. Jiaqiang Zhongguo Nongcun Pinkun Diqu Jiben weisheng Fuwu Xiangmu Wangong Zongjie Baogao [Final report on China Basic Health Services Project]. China Financial & Economic Publishing House; 2007.

刘运国、刘谷琮主编：《加强中国农村贫困地区基本卫生服务项目完工总结报告》，北京：中国财政经济出版社，2007年6月。

Policy documents

1. Central Committee of the Communist Party of China and State Council of China. Zhonggong Zhongyang Guowuyuan Guanyu Shenhua Yiyao Weisheng Tizhi Gaige de Yijian [Opinions of the CPC Central Committee and the State Council on Deepening the Health Care System Reform] [Internet]. Mar 17, 2009. Available from: http://www.china.org.cn/government/scio-press-conferences/2009-04/09/content_17575378.htm

《中共中央、国务院关于深化医药卫生体制改革的意见》（2009年3月17日）

1. Ministry of Civil Affairs of China, Ministry of Health of China, Ministry of Finance of China. Minzhengbu Weishengbu Caizhengbu Guanyu Shishi Nongcun Yiliao Jiuzhu de Yijian [Ministry of Civil Affairs, Ministry of Health and Ministry of Finance’s Opinions on the Implementation of Rural Medical Assistance]. 2003;158.

《民政部、卫生部、财政部关于实施农村医疗救助的意见》（2003年11月18日）

1. Central Committee of the Communist Party of China, State Council of China. Zhonggong Zhongyang Guowuyuan Guanyu Jin Yibu Jiaqiang Nongcun Weisheng Gongzuo de Jueding [Decision of the Central Committee of the Communist Party of China and the State Council to Strengthen Health Work in Rural Areas] [Internet]. Oct 19, 2002. Available from: http://www.gov.cn/gongbao/content/2002/content_61818.htm

《中共中央、国务院关于进一步加强农村卫生工作的决定》（2002年10月19日）

1. Central Committee of the Communist Party of China, State Council of China. Decision of the Central Committee of the Communist Party of China and the State Council Concerning Public Health Reform and Development [Internet]. Jan 15, 1997. Available from: http://www.asianlii.org/cn/legis/cen/laws/dotccotcpocatsccphrad1148

《中共中央、国务院关于卫生改革与发展的决定》（1997年1月15日）

Multimedia

1. China Central Television. CCTV-Yangguang Zhaojin Shangang [CCTV-The Sun Shone into the Hills] [Internet]. 2007 [cited 2022 Feb 5]. Available from: http://discovery.cctv.com/special/C18692/01/index.shtml

中国中央电视台：《阳光照进山岗》，央视一套《见证·亲历》，2007年6月16日至21日。

*HIV/AIDS Rolling Continuation Channel*

Project documents

1. Country Coordinating Mechanism, P. R. China. Proposal Form: Rolling Continuation Channel. Global Fund, 2008.
2. Wang X. Zhongguo Quanqiu Jijin aizibing xiangmu huigu he zongjie 中国全球基金艾滋病项目回顾和总结 [Summary of China Global Fund HIV/AIDS Program]. 2014.
3. Stirling M, Wang R, Jiang S et al. Strategic Review of Global Fund Experience in China: China AIDS Fund for Non-Governmental Organizations. Shehui Zuzhi Canyu Aizibing Fangzhi Jijin Wunian Jieduanxing Pinggu Baogao 社会组织参与艾滋病防治基金五年阶段性评估报告 [Five-Year Evaluation Report on the China AIDS Fund for Non-Governmental Organizations]., 2022.2003-2013., 2013.

Policy documents

1. State Council of China. Guowuyuan bangongting guanyu yinfa zhongguo ezhi yu fangzhi aizibing shisanwu xingdong jihua de tongzhi 国务院办公厅关于印发中国遏制与防治艾滋病“十三五”行动计划的通知 [State Council’s Notification on Issuing Twelfth Five Year Action Plan For HIV/AIDS Prevention and Control in China]. 2017.
2. State Council of China. Guowuyuan Bangongting Guanyu Yinfa Zhongguo Ezhi Yu Fangzhi Aizibing Shierwu Xingdong Jihua de Tongzhi 国务院办公厅关于印发中国遏制与防治艾滋病“十二五”行动计划的通知 [State Council’s Notification on Issuing Twelfth Five Year Action Plan For HIV/AIDS Prevention and Control in China]., 2012.

**Additional File 3. Each respondent’s profile**

| Case | Code | Level | Type of affiliation during the project | Engagement with the project |
| --- | --- | --- | --- | --- |
| Cross-case | R1-crosscase | International | World Health Organization | Project technical support |
|  | R2-crosscase | National | Ministry of Health | Project decision-making and management |
| Medical Financial Assistance in the World Bank–UK Basic Health Services Project (1998–2007) | R3-WB | National | Ministry of Health | Project decision-making and management |
|  | R4-WB | National | Ministry of Health | Project management |
|  | R5-WB | National | Ministry of Health | Project decision-making |
|  | R6-WB | National | Academic institute | Project’s and post-transition program’s decision-making, evaluation, and technical support |
|  | R7-WB | Provincial | Academic institute | Project’s and post-transition program’s decision-making, evaluation, and technical support |
|  | R8-WB | International | World Bank | All the project processes |
|  | R9-WB | International | World Bank | All the project processes |
|  | R10-WB  R11-WB | International | World Bank | Project decision-making and management |
|  | R12-WB  R13-WB  R14-WB  R15-WB  R16-WB | Provincial | Bureau of Health | Project’s and post-transition program’s decision-making and management |
|  | R17-WB | Provincial | Bureau of Health | Project management and post-transition program’s technical support |
|  | R18-WB  R19-WB | Prefectural | Bureau of Health | Project’s and post-transition program’s decision-making and management |
|  | R20-WB | Prefectural | Bureau of Health | Project’s and post-transition program’s decision-making, management, and technical support |
|  | R21-WB  R22-WB | Township | Health center | Project’s and post-transition program’s management and service delivery |
| Civil society engagement in the Global Fund HIV/AIDS Rolling Continuation Channel (2010–2013) | R23-GF | National | Center for Disease Control and Prevention | Project’s and post-transition program’s decision-making and management |
|  | R24-GF | National | Center for Disease Control and Prevention | Project decision-making and management and post-transition program’s technical support |
|  | R25-GF | Provincial | Center for Disease Control and Prevention | Project decision-making and management |
|  | R26-GF  R27-GF | National | Academic institution | Project’s and post-transition’s technical support |
|  | R28-GF | Provincial | Academic institution | Project’s and post-transition’s technical support |
|  | R29-GF | Provincial | Academic institution | Project implementation and post-transition program’s technical support |
|  | R30-GF  R31-GF | National | GONGO | Project decision-making and management |
|  | R32-GF  R33-GF  R34-GF | National | GONGO | Project’s and post-transition program’s decision-making and management |
|  | R35-GF | National | GONGO | Post-transition program’s decision-making and management |
|  | R36-GF | National | CSO | Project implementation and technical support |
|  | R37-GF | Cross-provincial | CBO | Project decision-making and post-transition program’s service delivery |
|  | R38-GF | Cross-provincial | CBO | Project decision-making and service delivery and post-transition program’s service delivery |
|  | R39-GF  R40-GF  R41-GF | Provincial | CBO | Project’s and post-transition program’s service delivery |
|  | R42-GF  R43-GF  R44-GF  R45-GF | Prefectural/District | CBO | Project’s and post-transition program’s service delivery |
|  | R46-GF | International | UNAIDS | Project management and technical support |

Abbreviations: ***CBO*** community-based organization; ***CSO*** civil society organization; ***GF*** Global Fund (the case); ***GONGO*** government-organized non-governmental organization; ***R*** respondent; ***WB*** World Bank (the case)

**Additional File 4. Examples of the interview guide**

An interview with a national MFA expert in the World Bank–UK Basic Health Services Project (1998–2007)

1. What is your position and role in the health sector? What was your involvement with the BHSP? What role did the national expert panel of MFA play in the national policy formulation on medical assistance?

2. How was the planning of the BHSP coordinated with the country health system and national health strategy? How did the project MFA interact with national medical assistance?

3. Sustainability plan: Was a sustainability plan developed?

If yes:

- By whom was it designed?

- How did it define sustainability?

- What did it plan sustainability of interventions supported by the project after the project ends? (Particularly for MFA)

4. Transition period: did the project have a “transition” period, especially MFA?

If yes:

- What criteria were used to define the transition period?

- How were those negotiated? - By whom?

- How was the timing decided and by whom?

- How was transition designed?

- Who was involved in the transition planning process? Who were the stakeholders engaged in the decision to transition donor funding? Was there involvement across sectors?

- Were those expected to take over the project activities, involved in the transition design and implementation?

- Who were actors engaged in either the implementation of the transition policy/supported programs and what were their roles?

- What roles did government, civil society, and development partners play in this process? How did this influence the evolution of the project’s content?

- How did the transition process influence what was finally transitioned in terms of each health system function and how and why did institutions take over or not take over the functions initially envisaged?

- How did the transition process prepare key actors for the adaptation of functions previously supported by external funding? Did this include a concerted effort to build the capacity of national institutions?

5. Governance

- What was the level of commitment from the political level for the project? Did this vary between the pre and post transition periods?

- What was the budgeting process for the project?

- Was there a donor coordination arrangement that worked with the MoH? What role did this play, and how did this vary pre and post transition?

- What’s the role of project management office in transition?

- What were the accountability mechanisms within the project?

6. Financing

- What was the source of funding for this project at the starting point? Was there more than one source? If so in what proportion were different sources providing funding? How did the funding source/s of different components change post transition?

- To what extent have financial responsibilities been transferred from World Bank/DFID to China?

- How was domestic financing for the intervention scaled up? What is the balance between domestic government funds and out-of-pocket payments?

- To what extent has the entity implementing the project (esp. the subnational one) post transition secured adequate funding to sustain interventions?

- To what extent were the budgetary and financial systems of the project pre transition, aligned with those of China? How did this evolve over time?

7. Inputs

- To what extent were there technical, managerial, and financial capacities within China to effectively deliver key health program services? How does this compare between the pre-transition and post transition periods?

- Were consumables and pharmaceuticals for the project procured, stored and distributed through dedicated supply chains or did the project use general domestic systems? How was this different between the pre and post transition periods?

- How were data generated, managed and used by the project? Were information systems for the project separate or were they integrated with broader health information systems? How was this different in the pre and post transition periods?

- To what extent were information systems actively used and by whom to make decisions such as those around human resource management, supply of drugs and other consumables and delivery of services for example. How did this vary across the pre and post transition periods?

- To what extent did the projects M&E systems align with China’s M&E systems, including indicators and reporting periods? How did this change between the pre transition and post transition periods?

- What kind of support for human resources for health was present in the project pre-transition? How did this change post transition?

- Did the project have dedicated human resources? How did the project compensate HR? Did this vary between pre and post transition periods?

- To what extent were reporting structures for human resources in the project pre-transition aligned with those of China? How did this evolve during the process of transition and after transition?

9. Transition results and factors

- What do you think of transition results of the BHSP, especially medical financial assistance? How have key outcome indicators and health outcome indicators relating to the donor-supported interventions changed? How has the effective coverage of project-supported interventions changed after transition? What is the impact of project transition on the crude coverage of services previously covered by external funding?

- How did intervention, donor and recipient characteristics influence transition?

- How did political, institutional, economic, socio-cultural context influence transition?

10. Others

- What’s your overall observation on transition of external assistance in China?

- Who would you recommend as the key informant(s)?

- Any documents or data sources recommended?

An interview with a subnational CBO respondent in the HIV/AIDS Rolling Continuation Channel supported by the Global Fund to Fight AIDS, Tuberculosis and Malaria (2010–2013)

1. What was your role in Global Fund projects and in RCC? How were you involved in it? (project design, implementation, evaluation, etc.)

- What projects and in which province(s)?

2. We are also interested in the general picture of Global Fund and AIDS in China. What’s your impression of the socio-political context of AIDS and international development cooperation during the Global Fund years, especially during 2010-13?

- Were there any observable changes before and after GF?

2. Is there any specific focus, say, in any areas or groups of people, of RCC projects in V [anonymous province]? Is it different from previous rounds?

3. In your experience or observation, what was the status of development like for NGOs during RCC years (in V [anonymous province])?

- What types of NGOs that were in the play then?

4. How was the RCC ended? Is your organization or projects affected in any form? As you may know, RCC was supposed to last till 2015 but was, in fact, ended two years earlier.

5. So far what other international donors or development partners you have worked with in AIDS projects? How are they different from the Global Fund?

6. Do international partners act differently in transition? How?

7. Comparatively speaking, what means of transition, or simply for example which project, do you consider to be more sustainable in the local context?

8. All projects will come to an end. Were there any considerations or discussion in V [anonymous province] over the sustainability of GF projects? Are there any concrete sustainability plans?

9. Is there a “transition” period as the GF wraps up and the health services originally supported by them transferred onto the hand of local governments and became a routine part of the health system?

- When was this period?

- Was there any disruption, either temporary or permanent, especially related to NGOs? Such as in funding, capacity building, etc.

- In terms of NGOs, which governmental body or organization took over their management?

10. We know that the Government Procurement of Services from NGOs in AIDS prevention and control started in 2012 in V [anonymous province] and later in other provinces. Are you familiar with this – how it was planned and started?

- Has your organization ever participated in the China AIDS Fund for NGOs?

- How has your organization been working with local government?

11. Who were involved in the transition planning process?

- Who were the stakeholders engaged in the decision to transition donor funding?

- What were the roles of local governments, the Chinese Association of STD & AIDS Prevention and Control, NGOs, etc.?

12. How was the decision to transition communicated?

13. As Global Fund was leaving China, were there any efforts or plans, such as training, to strengthen the capacity building for NGOs?

14. As a vertical HIV/AIDS project, how did RCC coordinate with V’s [anonymous province] local health system, or strengthen its capacity building, so as to ensure GF projects’ sustainability?

15. What kinds of interventions were conducted during RCC? Were there any changes post transition? How about HPP?

- Changes in terms of quality, coverage of groups of people, service providers, etc.

16. Is there a difference regarding the quality of intervention conducted by NGOs or the government?

- Will the underdevelopment of NGOs in post-RCC years lead to quality drop in interventions?

17. What was the level of commitment from the government for AIDS? Did this vary between the pre and post transition periods?

18. What was the coordination mechanism in Global Fund like? What role did this play, and how did this vary pre and post transition?

- #what’s the role of project office in transitioning?

- Are methods of bidding, project coordination, evaluation that were used in GF years still being used?

19. On a routine basis, how are NGOs being managed in V [anonymous province]? How do they coordinate with the government?

- Any cross-departmental mechanism?

20. What were the accountability mechanisms within the project? - How were these accountability mechanisms used?

- Were there any significant differences with the rest of the health system? How did this vary pre and post transition?

21. How did the source of funding change? And for those that haven’t been legally registered?

- How was domestic financing for the intervention scaled up? Is the Chinese government taking up full financial responsibility after the Global Fund left?

- What about financial transparency?

22. Inputs

- To what extent were there technical, managerial, and financial capacities within China to effectively deliver key health program services? How does this compare between the pre-transition and post transition periods?

- Were consumables and pharmaceuticals for the project procured, stored and distributed through dedicated supply chains or did the project use general domestic systems? How was this different between the pre and post transition periods?

- How were data generated, managed and used by the project? Were information systems for the project separate or were they integrated with broader health information systems? How was this different in the pre and post transition periods?

- To what extent were information systems actively used and by whom to make decisions such as those around human resource management, supply of drugs and other consumables and delivery of services for example. How did this vary across the pre and post transition periods?

- To what extent did the projects M&E systems align with China’s M&E systems, including indicators and reporting periods? How did this change between the pre transition and post transition periods?

- What kind of support for human resources for health was present in the project pre-transition? How did this change post transition?

- Did the project have dedicated human resources? How did the project compensate HR? Did this vary by facility type (public, NGO, private) or level of facility (primary, secondary, tertiary)? Did this vary between pre and post transition periods?

- To what extent were reporting structures for human resources in the project pre-transition aligned with those of Chinas? How did this evolve during the process of transition and after transition?

23. In general, how do you think of the transition outcomes of RCC? Specifically, which aspects do you consider successful and which not?

- How have key outcome indicators and health outcome indicators relating to the donor-supported interventions changed?

- How has the effective coverage of project-supported interventions changed after transition? Why?

24. What are the enabling and impeding factors that can lead to a successful, sustainable transition?

25. In conclusion, do you think the Global Fund has positioned and steer the development of Chinese NGOs in AIDS to a right direction? Has it carried through such value or belief in civil society engagements despite pushbacks from the Chinese government?

26. As China graduates as a recipient country and becomes an emerging donor, what lessons learned, or inspiration are there from the Global Fund project?

27. Any documents/data sources to recommend? Who would you recommend as the key informant(s) of the RCC Project, especially those worked in V [anonymous province]?
